# Target Capture Sequencing Provides Insights Into Hybridogenetic Water Frogs

**DOI:** 10.1002/ece3.73570

**Published:** 2026-04-29

**Authors:** Lisanne van Veldhuijzen, Maciej Pabijan, Tariq Stark, Richard P. J. H. Struijk, Ben Wielstra, James France

**Affiliations:** ^1^ Institute of Biology Leiden Leiden University Leiden the Netherlands; ^2^ Naturalis Biodiversity Center Leiden the Netherlands; ^3^ Department of Comparative Anatomy, Institute of Zoology and Biomedical Research, Faculty of Biology Jagiellonian University Kraków Poland; ^4^ Reptile, Amphibian and Fish Conservation Netherlands (RAVON) Nijmegen the Netherlands

**Keywords:** amphibians, hybridization, sequence capture, triploid

## Abstract

The European water frogs of the genus *Pelophylax* present a genetic puzzle that has not yet been studied with strong genomic tools. This limitation has hampered investigation into, for example, the remarkable hybridogenetic system observed in the genus, as well as the monitoring of invasive populations—a critically vulnerable threat to *Pelophylax*. In this study we explore whether a previously developed target capture bait set, FrogCap, can be used to answer genomic questions within the genus by applying it to the three taxa of *Pelophylax* native to the Netherlands: the marsh frog *P. ridibundus* , the pool frog *P. lessonae* , and their hybridogenetic hybrid, the edible frog *P. esculentus* . We ask if we can obtain high coverage genomic data, distinguish the different species, identify ploidy level, and detect putative interspecific nuclear gene flow. We observe that the high‐quality target capture data obtained cleanly separates the three taxa, confirming significant introgression of *P. lessonae* mtDNA into *P. ridibundus* from the Netherlands. We also show that target capture is an efficient method for identifying polyploidy in *P. esculentus* and is effective at determining ancestral contribution within our sample population. Targeted sequence capture using FrogCap is a useful tool to unravel the intricate evolution of *Pelophylax* water frogs.

## Graphical abstract

The genomics of European frogs of the genus *Pelophylax* are intriguing due to their propensity to hybridise, the prevalence of polyploidy, and the unusual hemiclonal hybridogenetic complexes formed in many populations. This study examines the use of target capture sequencing using the Frog Cap bait set to investigate these complex systems. Target capture sequencing proves an effective tool for population genomics in this genus.

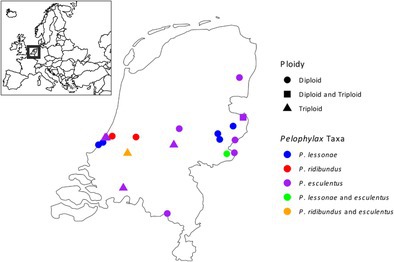

## 1 Introduction

The edible frog, *Pelophylax esculentus*, is one of the most prominent examples of an extraordinary mode of reproduction known as hybridogenesis (Dufresnes and Mazepa 2020; Lavanchy and Schwander 2019). *Pelophylax esculentus* is the fertile hybrid of the pool frog, *P. lessonae* , and the marsh frog, *P. ridibundus* , which are sympatric across a large region of Europe (Berger 1967; Dufresnes and Mazepa 2020). While *P. esculentus* carries the genomes of both of its parents, these do not undergo recombination with each other and the hybrid will only transmit the genome of one species to its offspring (unless the hybrid is polyploid) (Graf and Polls Pelaz 1989; Uzzell et al. 1976; Uzzell and Berger 1975). Generally, the same parental genome is transmitted for many generations, resulting in a clonally transmitted lineage that reproduces itself at the expense of a sexual host species. This behavior results in *P. esculentus* being designated as a klepton (and sometimes abbreviated as *P*. kl. *esculentus*) (Dubois and Günther 1982).

Due to the lack of recombination between the parental genomes, all *P. esculentus* are effectively F_1_ hybrids, even though they may be descended from many generations of backcrossing. Consequently, *P. esculentus* individuals share a similar set of characteristics, intermediate between the parental species, and so may be considered as a distinct taxon. The morphology of *P. lessonae* and *P. ridibundus* varies considerably across their range, making distinguishing the three taxa difficult in some areas (Arntzen 1981; Meilink et al. 2024). However, they retain distinct ecological preferences and mating calls (Roesli and Reyer 2000).

Variation exists regarding the specific parental genome transmitted. In the most well‐known example, *P. esculentus* occurs in mixed populations with *P. lessonae* , referred to as the *lessonae‐esculentus* (or L‐E) system, where *P. esculentus* selectively transmits only the *P. ridibundus* genome. In other populations the situation is essentially reversed to form the *ridibundus‐esculentus* (R‐E) system (Hoffmann et al. 2015; Holsbeek and Jooris 2010; Uzzell and Berger 1975). In either case, *P. esculentus* could theoretically mate with itself to regenerate the alternative parental species. However, the offspring of matings between two *P. esculentus* are rarely viable (Berger 1967; Guex et al. 2002; Vorburger 2001). This is presumed to be due to such offspring inheriting two copies of the clonal genome. As this genome never undergoes recombination, it is unable to efficiently purge deleterious recessive alleles, leading to the phenomenon known as Muller's ratchet (Muller 1964). In cases where two *P. esculentus* from different clonal lineages mate, offspring viability is notably higher (Guex et al. 2002; Vorburger 2001).

In addition to the L‐E and R‐E systems, populations exist that include all three taxa (the L‐E‐R system), as well as populations containing solely *P. esculentus* (the E–E system) (Hoffmann et al. 2015). The existence of the E‐E system is associated with polyploid individuals, which also allow for recombination within the clonal genome. Furthermore, these systems also involve the production of unreduced gametes, which transmit both parental genomes, preserving hybridity (Christiansen 2009; Christiansen and Reyer 2009; Dubey et al. 2019). Polyploidy is not exclusive to the E‐E system and may be observed across the range of *P. esculentus* (Biriuk et al. 2016; Blommers‐Schlösser 1990; Christiansen and Reyer 2009; Dedukh et al. 2022).

A significant body of research has focused on the cytology of hybridogenesis in *P. esculentus* . It is observed that within the gonocytes of tadpoles, the chromosomes forming one of the parental genomes are corralled into micronuclei and gradually degraded, whereas the other parental genome is duplicated prior to meiosis (Chmielewska et al. 2018; Dedukh et al. 2020). However, there has been comparatively little study of the genomics of the hybridogenetic system. Molecular investigation in *Pelophylax* has largely focussed on allozymes (Hotz et al. 2013; Mezhzherin et al. 2024) and limited numbers of nuclear and mitochondrial markers (Dufresnes, Monod‐Broca, et al. 2024; Papežík et al. 2024; Patrelle et al. 2011; Sagonas et al. 2020). A small number of RAD‐sequencing based studies provide wider genomic data (Doniol‐Valcroze et al. 2021; Dubey et al. 2019; Dufresnes and Dubey 2020), but as RAD‐sequencing cannot be targeted to specific loci (e.g., coding genes), the resulting datasets are difficult to integrate due to frequent gaps and non‐overlapping marker sets.

The lack of genomic data presents a major barrier to understanding the evolution of hybridogenesis in *Pelophylax* and the molecular mechanisms behind it. Presumably genetic or epigenetic factors determine whether the *lessonae* or *ridibundus* genome is transmitted, however no candidate loci are known. In principle the hybridogenetic system should exclude the possibility of nuclear DNA transfer between the parental species (as one of the genomes is eliminated prior to meiosis). However, the system has been observed to be subject to a degree of lability (Biriuk et al. 2016; Dedukh et al. 2019). Allozyme and AFLP data suggest that nuclear introgression between parental species via *P. esculentus* can occur in certain populations (Mezhzherin et al. 2024; Mikulíček et al. 2014; Uzzell et al. 1976). More comprehensive genomic data would be essential to determine if such interspecific gene flow is taking place.

Genomic data may also be used to distinguish *Pelophylax* species. However, molecular species identification in *Pelophylax* is complicated because *P. esculentus* has no mitochondrial DNA of its own, and mtDNA introgression between the parent species is common in some areas (Plötner et al. 2008). Nuclear markers have the potential to resolve this, but the commonly used marker SAI‐1 is not entirely reliable (Dufresnes, Dubey, et al. 2024), and there is a lack of other sequences with known species‐specific variants.

Target capture sequencing allows for hundreds to thousands of markers to be sequenced in each sample via hybridization with pre‐designed RNA baits (Gnirke et al. 2009). As the targeted markers are chosen in advance, it is simple to integrate data from multiple studies. Generally coding DNA and other highly conserved sequences are chosen as markers, resulting in data which is more interesting from a functional perspective and more suitable for comparison between widely divergent lineages (Andermann et al. 2020). Additionally, target capture data can be used to determine the ploidy of individuals (particularly in hybrids) (Weiß et al. 2018).

Target capture requires baits to be designed in advance, however as the method is amenable to significantly divergent sequences, it is often feasible to use a bait set designed for one taxon in related taxa or design a bait set covering an extremely large number of species (de Visser et al. 2025; Yardeni et al. 2022). Recently FrogCap, a bait set designed for use across the order Anura, has been successfully used in several studies (Chan et al. 2020; Hutter et al. 2022; Rasolonjatovo et al. 2020). Here we aim to test the usefulness of FrogCap for investigation of the genomics of the taxa comprising the *P. esculentus* hybridogenetic complex.

We gather *Pelophylax* samples from across the Netherlands, including all three taxa: the marsh frog *P. ridibundus* , the pool frog *P. lessonae* , and their hybridogenetic hybrid, the edible frog *P. esculentus* . We also include samples from Poland—where the morphology of the taxa appears to be more distinct than in the Netherlands (Arntzen 1981; Berger 1973). We then use the FrogCap bait set to explore whether (1) it is possible to obtain high coverage genomic data from *Pelophylax* samples, (2) the species can be accurately determined from the molecular data, (3) the ploidy of the genomes can be identified, and (4) any nuclear gene flow between *P. lessonae* and *P. ridibundus* can be detected.

## 2 Methods

### 2.1 Samples

We used samples from a total of 45 individuals in this study, including 38 samples originating from the in the Netherlands (obtained from 19 localities consisting of 18 buccal swabs, 13 skin swabs and 7 tissue samples) and previously reported in Theodoropoulos et al. (2025). Seven samples were obtained from six localities within Poland; these consisted of toe clips or muscle collected with permission from the General Directorate for Environmental Protection (permit nos. DZP‐WG.6401.02.2.2018.kp.2 and DZP‐WG.6401.75.2023.TŁ.2). The total set of 45 samples included 11 *P. lessonae* , 22 *P. esculentus* , and 12 *P. ridibundus* . Previous barcoding showed that with the exception of one *P. esculentus* all samples from the Netherlands possess *P. lessonae* mtDNA (Theodoropoulos et al. 2025). The seven Polish samples were mitotyped using a PCR‐based method that uses *lessonae*‐ and *ridibundus*‐specific primers to amplify fragments of mtDNA that can be verified on an agarose gel (Jośko and Pabijan 2021). Data for all samples can be found in Table S1.

### 2.2 Library Preparation

Concentration of DNA extracts was standardized to a minimum of 100 ng/μl. Library preparation was performed using the NEBNext Ultra II FS DNA Library Prep Kit for Illumina (New England Biolabs, MA, USA), using the manufacturer's instructions with all volumes divided by four. DNA was fragmented enzymatically for 6:30 min before adapter ligation. Size selection targeting a 300 bp insert size was performed using NucleoMag beads (Macherey‐Nagel, Düren, Germany). Unique combinations of custom i5 and i7 index primers (Integrated DNA Technologies, Leuven, Belgium) were incorporated via nine cycles of PCR. Library concentration and quality were assessed using the Agilent 4200 Tapestation system (Agilent, CA, USA). After library preparation, libraries were pooled equimolarly in batches of 15 with 250 ng of DNA per sample, and vacuum concentrated to 800 ng/μL.

### 2.3 Target Capture Sequencing

Target enrichment capture was performed with the FrogCap Ranoidea‐v2 marker set (Hutter et al. 2022) (serial code D10260HFRn) using the Mybaits V 5.0 kit (Arbor Biosciences, MI, USA). After a 30 min pre‐hybridisation incubation with the blocking mix, pooled libraries were hybridized at 63°C for 24 h. Enriched libraries were bound to streptavidin coated beads and washed to remove non‐target DNA before being subjected to 14 cycles of PCR using the KAPA HiFi Master Mix (Roche, Basel, Switzerland). 150 bp paired‐end sequencing was performed using the NovaSeq 6000 platform (Illumina Inc., San Diego, CA, USA) by BaseClear B.V. (Leiden, Netherlands), targeting 2 Gbp of sequencing data per individual.

### 2.4 Processing of Sequence Capture Data

We used a bioinformatics pipeline adapted from the NewtCap protocol (de Visser et al. 2025). The upstream data processing was performed via a custom Perl (version v3.38.0) script (*Pipeline_1.pl*). Raw demultiplexed reads were trimmed with Trimmomatic (version 0.39) (Bolger et al. 2014) and BBDuk version 38.96 (Bushnell et al. 2017) to remove adapter contamination. BWA‐MEM (version 0.7.17) (Li 2013) was used to map the trimmed reads against reference assembly consisting of the FrogCap Ranoidea‐v2 consensus marker sequences (Hutter et al. 2022). The resulting BAM files were processed, deduplicated and genotyped via the *AddOrReplaceReadGroups*, *MarkDuplicates* and *HaplotypeCaller* functions of GATK (version 4.5.0.0) (McKenna et al. 2010). The resulting VCF files were jointly genotyped with the *GenomicsDBImport* and *GenotypeGVCFs* function of GATK.

Sequencing depth was assessed with a custom R (version 4.4) (R Core Team 2024) script (*Peakloop2.R*) which evaluated the minimum depth of the best covered continuous 100 bp sequence within each target sequence of the reference assembly (this metric was chosen as it is not affected by reference assembly target length). For each individual, the coverage of each marker was collected, and median and total values were calculated. A graph was produced to visualize the performance of each marker in Rstudio (version 24.04.2) (Posit team 2025) using the packages ggplot2, patchwork and tidyr (Pedersen 2025; Wickham 2016; Wickham et al. 2025). The median coverage of each marker was calculated and markers were then ordered by the sum of their median coverage.

The joint VCF file was filtered using VCFtools (version 0.1.16) (Danecek et al. 2011) to remove indels, sites with a minor allele frequency of less than 0.1 and sites with missing data, sequencing depth of less than 10, or genotype quality of less than 20, in any individual. Finally the *–thin* function was used to select one SNP per reference sequence.

### 2.5 Principal Component Analysis

Principal Component Analyses (PCAs) were performed using the R packages gdsfmt and SNPRelate (Zheng et al. 2012). An initial PCA was performed on the entire dataset (all 45 samples) and then separate PCAs were performed on each of the three taxa targeted in this study (11 *P. lessonae* , 22 *P. esculentus* , and 12 *P. ridibundus* ). The results of the analysis were visualized with the ggplot2 package (Wickham 2016).

### 2.6 Admixture Analysis

ADMIXTURE (Alexander et al. 2009) was used to determine the ancestry of each sample. Input consisted of a VCF file containing one random SNP per marker, filtered for sites with no missing data. As *P. esculentus* is a hybrid of the two other taxa involved in this study, the number of ancestral gene pools (K) was set to two (as there are only two nuclear lineages of interest within the study population). Twenty‐five replicates of the analysis were combined with the program CLUMPAK (Kopelman et al. 2015) and the results were visualized with the ggplot2 package (Wickham 2016).

### 2.7 Heterozygosity/Ancestry Analysis

After importing VCF data with the vcfR package (Knaus and Grünwald 2017), the triangulaR (Wiens et al. 2025) package was used to calculate the hybrid index and interclass heterozygosity based on SNPs that were 100% diagnostic between the parental species (i.e., SNPs that were fixed in one state in all 11 *P. lessonae* individuals and as another state in all 12 *P. ridibundus* individuals). The data was then visualized with the ggplot2 package (Wickham 2016). The hybrid index reflects the proportion of ancestry from either one or the other parental group and the interclass heterozygosity the proportion of markers that are heterozygous (Fitzpatrick 2012). Therefore, given that we employ SNPs that show fixed differences between the parentals, a pure parental should have an interclass heterozygosity of 0 and a hybrid index of either 0 or 1, and an F1 hybrid should have an interclass heterozygosity of 1 and a hybrid index of 0.5. Potential later generation hybrids such as F2s and backcrosses have different theoretical expectations of hybrid index and interclass heterozygostisy (see also figure 1 in Wiens et al. 2025).

### 2.8 Ploidy Analysis

The species‐specific SNPs used to determine hybridity were also employed to determine the ploidy and parental contribution of the *P. esculentus* samples. For each SNP locus in each sample, the ratio of the sequencing depth of the *P. lessonae* allele to the total sequencing depth was calculated. For diploid *P. esculentus* samples, these ratios should average 0.5; for triploids, the ratios should be either 0.33 or 0.66, depending on whether the individual possesses an extra copy of the *P. ridibundus* or *P. lessonae* genome. For each sample, the frequency of SNPs was plotted against the calculated allele ratio.

Additionally, the ploidy of all samples was assessed using the program nQuire (cloned from Git commit 8975c94) (Weiß et al. 2018). The BAM files produced for each sample were used as input to create .bin files via the *create* function with ‐c set to 20 and ‐p to 20. The *denoise* function was then used on the .bin files, followed by ploidy model fitting with the *histotest* and *view* functions. The resulting allele ratio distributions were normalized and combined. Gaussian mixture models were utilized to assess the ploidy level, where a free model is compared to models fixed on diploidy, triploidy, and tetraploidy. The model with the lowest difference from the free model was interpreted as the most likely ploidy of the sample. The results were visualized with the ggplot2 package (Wickham 2016).

## 3 Results

### 3.1 Sequence Capture Performance

We obtained 134.9 Gbp of raw sequence data, with a mean of 11,074,445 (s.d. 5,009,066) read pairs per sample. A mean of 69.4% (s.d. 8.6%) are duplicates and 37.2% (s.d. 15.2%) of reads can be mapped to the target sequences, resulting in a mean of 11.4% of read pairs being useful (1,260,626 read pairs per sample). After deduplication and mapping, median peak coverage varies between samples from a minimum of 9 to a maximum 81 (mean 35.4, s.d. 23.4). Coverage varies between target sequences. Across all samples, 2395 of 12,909 markers (18%) have a median coverage across all samples of over 100, and 4407 markers (34%) have less than 10. VCF filtering resulted in a final selection of 1957 SNPs available for further analysis.

### 3.2 Principal Component Analysis

**FIGURE 1:**
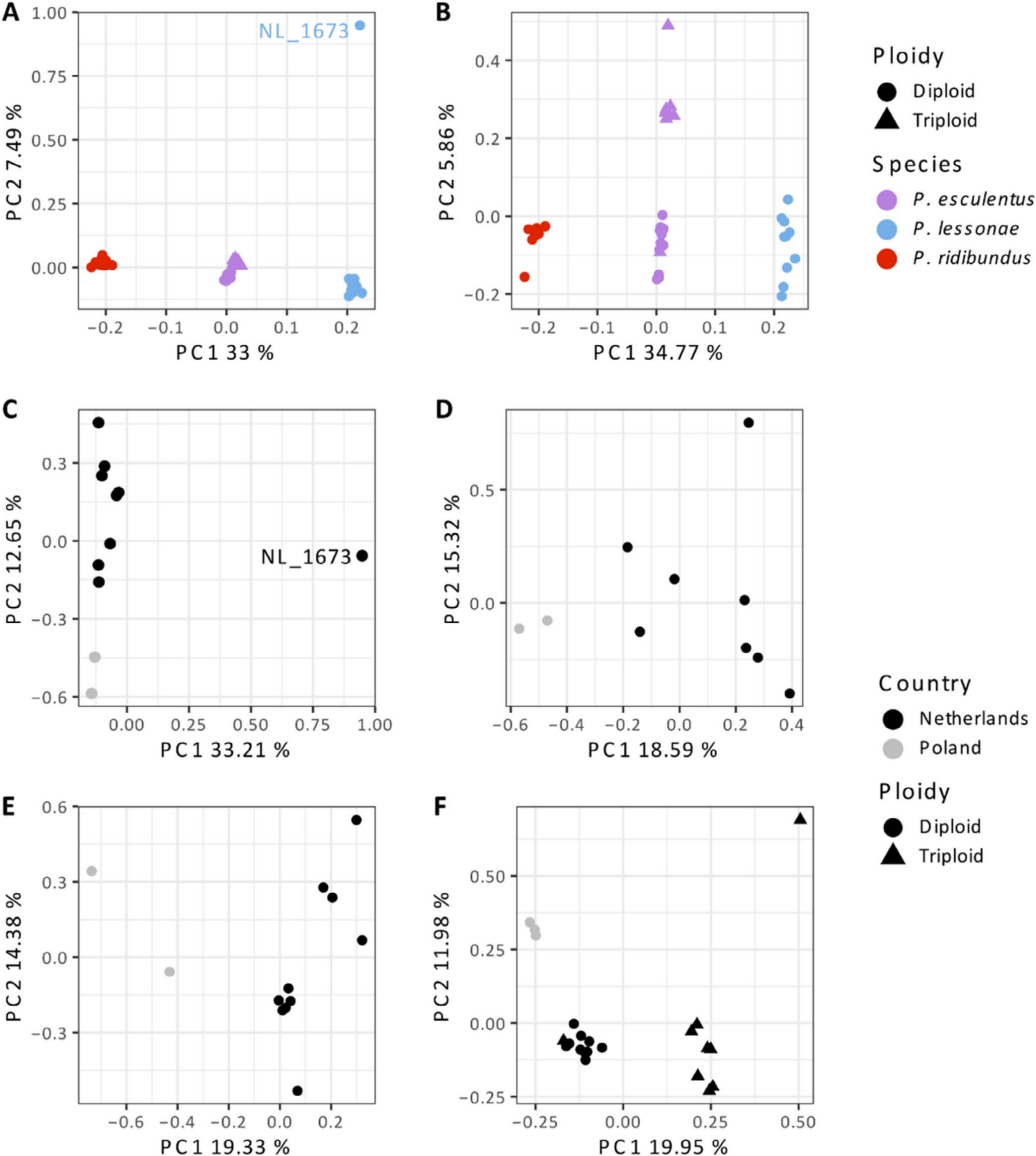
Principal Component Analysis of waterfrogs (*Pelophylax*). (A) PCA including all samples used in this study, the three taxa are separated by the first principal component. A single *P. lessonae* (NL_1673) accounts for the major variation along the second principal component, with all other samples tightly clustered by taxa. (B) PCA including all samples other than NL_1673, the first principal component is almost unchanged, but the taxa diverge along the second principal component. (C) All *P. lessonae* samples. (D) *P. lessonae* excluding NL_1673. (E) all *P. ridibundus* samples. (F) All *P. esculentus* samples.

In the PCA including all 45 samples (Figure 1A), three distinct groups are separated by the first principal component (PC1), which explains 33% of the total variation in the dataset. PC1 corresponds to *lessonae*‐*ridibundus* variation, with all *P. esculentus* samples aligning near the origin of this axis, and all *P. lessonae* and *P. ridibundus* located in discrete clusters equidistant at both sides from the origin. A single *P. lessonae* individual (NL_1673) is the only sample to vary significantly along the PC2 axis (which accounts for 7.5% of total variation). As this sample has excellent coverage and was worked up together with the other samples, a technical reason for it being an outlier appears to be unlikely. To reveal any variation obscured by this sample, we repeat the PCA with it excluded (Figure 1B), but this does not show a significant effect on the overall clustering of samples. When separate PCAs (Figure 1C–F) are performed for each taxon, we observe that the Polish individuals form a distinct cluster in all three cases. We also observe a clustering of triploid vs. diploid samples among Dutch *P. esculentus* (see the ploidy analysis below).

### 3.3 Admixture Analysis

**FIGURE 2:**
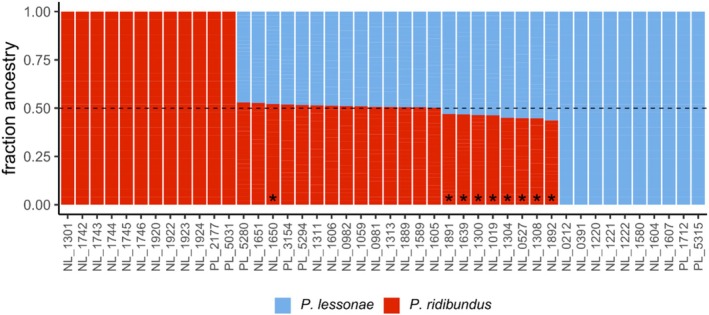
Admixture analysis of waterfrogs (*Pelophylax*). Triploid samples are denoted with an asterisk (*). All samples have either 100% ancestry from one of the parent species, or close to 50% ancestry from both. The triploid *P. esculentus* samples that show slightly higher *P. lessonae* contribution all have two copies of the *P. lessonae* genome, whereas the remaining triploid has two copies of the *P. ridibundus* genome.

All *P. ridibundus* and *P. lessonae* individuals have complete *P. ridibundus* and *P. lessonae* ancestry, respectively (Figure 2). All *P. esculentus* samples show a mixed ancestry, however in several the contribution from *P. lessonae* is slightly greater than the expected 50%. This is particularly the case in triploid samples that carry two copies of the *P. lessonae* genome.

### 3.4 Ancestry/Heterozygosity Analysis

**FIGURE 3:**
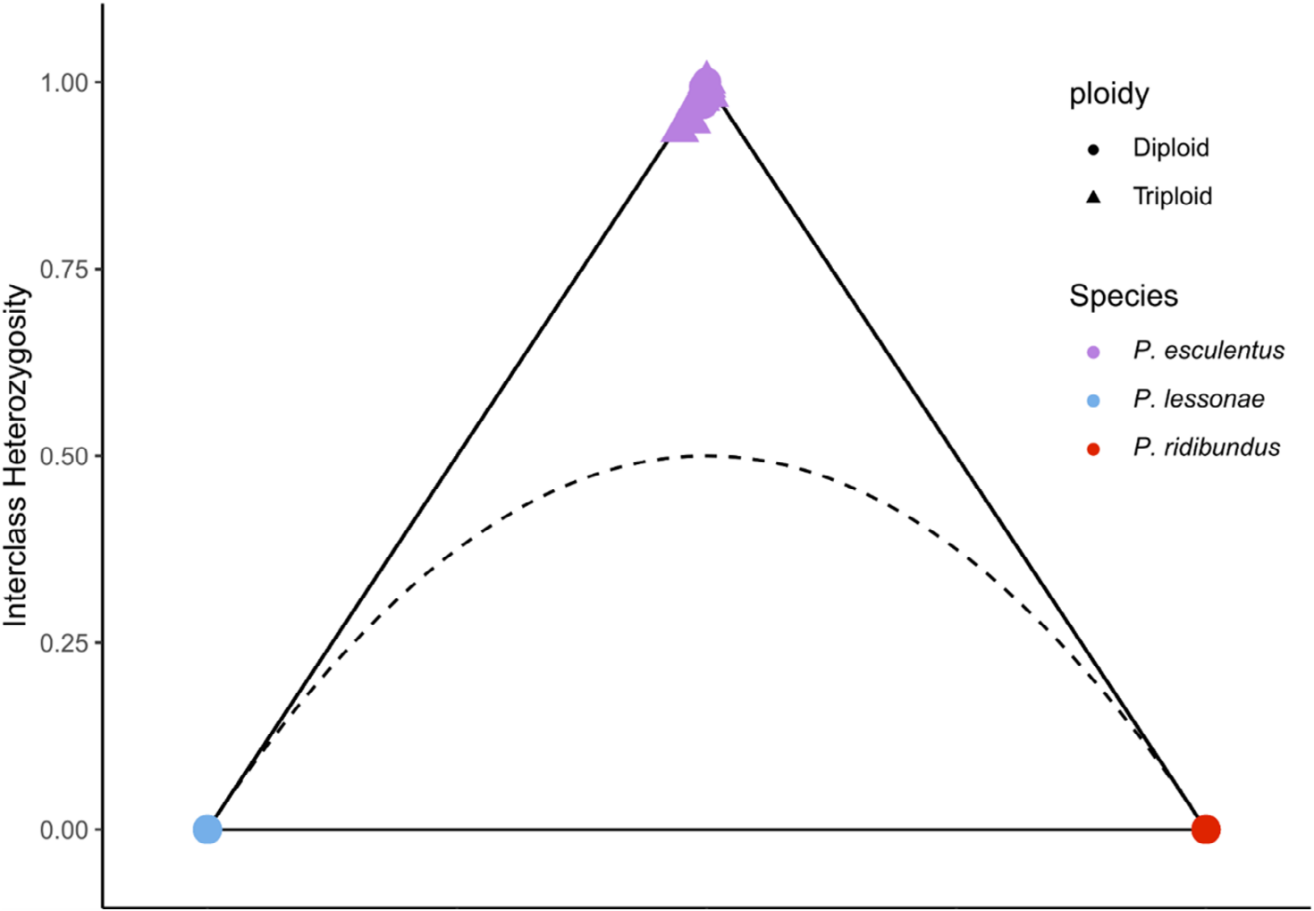
Heterozygosity/hybridity analysis of waterfrogs (*Pelophylax*). The hybrid index (x‐axis) indicates what percentage of the markers of an individual belong to either parent species (with markers with alleles from both parental species being scored as 0.5, regardless of the actual allele ratio), where 0 represents *P. lessonae* and 1 *P. ridibundus* . The y‐axis indicates the interclass heterozygosity, meaning the proportion of loci with alleles from both parental groups. The dotted black curve indicates the boundary below which individuals cannot occur, assuming Hardy–Weinberg Equilibrium. All *P. esculentus* samples behave similarly to an ideal F1 hybrid (with all markers being heterozygous and having 50% ancestry from each parental species).

696 SNPs were 100% species diagnostic in all individuals from the parental species. All *P. esculentus* samples cluster in the upper vertex of the triangle plot (Figure 3) – with a hybrid index close to 0.50 and an interclass heterozygosity close to 1. This is the expected behavior of F1 hybrids. However, corresponding to the admixture analysis some *P. esculentus* , particularly triploids with two copies of the *P. lessonae* genome, are slightly displaced in the direction of *P. lessonae* , with NL_1304 and NL_1892, the triploids with the lowest coverage, showing the greatest displacement.

### 3.5 Ploidy Analysis

**FIGURE 4:**
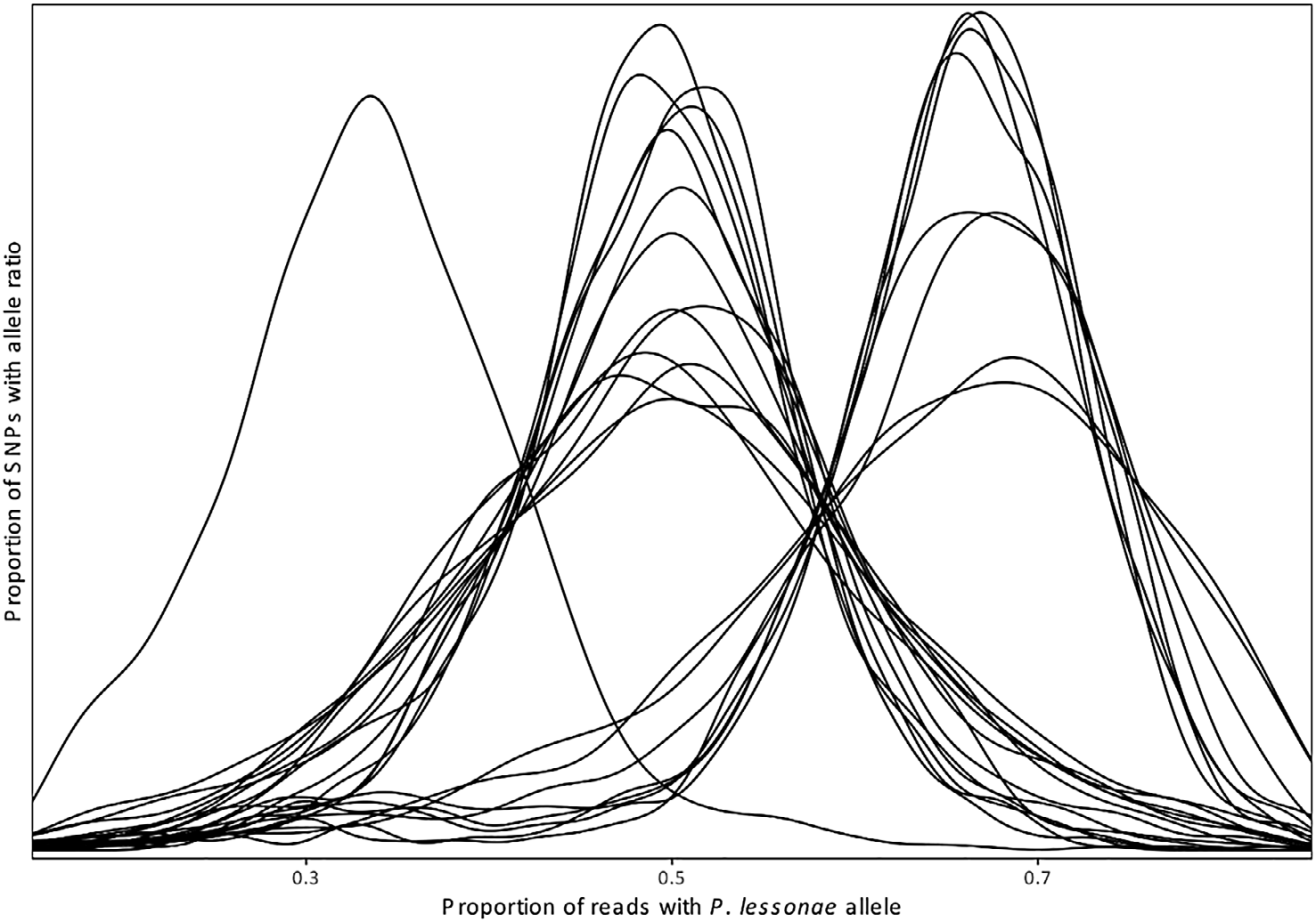
Polyploidy and genome composition of *Pelophylax esculentus* samples. For diploid samples the proportion of reads with *P. lessonae* specific SNPs is normally distributed around 0.5. Eight samples instead show a mean ratio of approximately 0.66, indicating that they are triploid with two copies of the *P. lessonae* genome and a single copy of the *P. ridibundus* genome. A single sample has the opposite genome composition, as indicated by its peak at approximately 0.33.

We observe that 13 of the 22 *P. esculentus* samples show allele ratios normally distributed around 0.5 for the SNPs diagnostic for the parent species, typical of diploidy (Figure 4). Of the nine remaining *P. esculentus* samples, eight show allele ratios of 2:1 in favor of *P. lessonae* , indicating that they are triploids with two copies of the *P. lessonae* genome. The remaining sample is also triploid but with allele ratios 2:1 in favor of *P. ridibundus* .

Analysis of the *P. esculentus* data with nQuire confirms the samples consist of nine triploids and 13 diploids. All *P. lessonae* and *P. ridibundus* samples are diploid (Figures S1–S3). The number of markers available for ploidy analyses varies with both overall coverage and heterozygosity, resulting in lower *R* ^2^ values for the parental species (which are less heterozygous than the parents). However, while the *P. lessonae* and *P. ridibundus* samples typically have fewer than half the informative markers compared to *P. esculentus* samples, there is enough data available for confident ploidy determination in all samples.

### 3.6 Geographic Pattern

**FIGURE 5:**
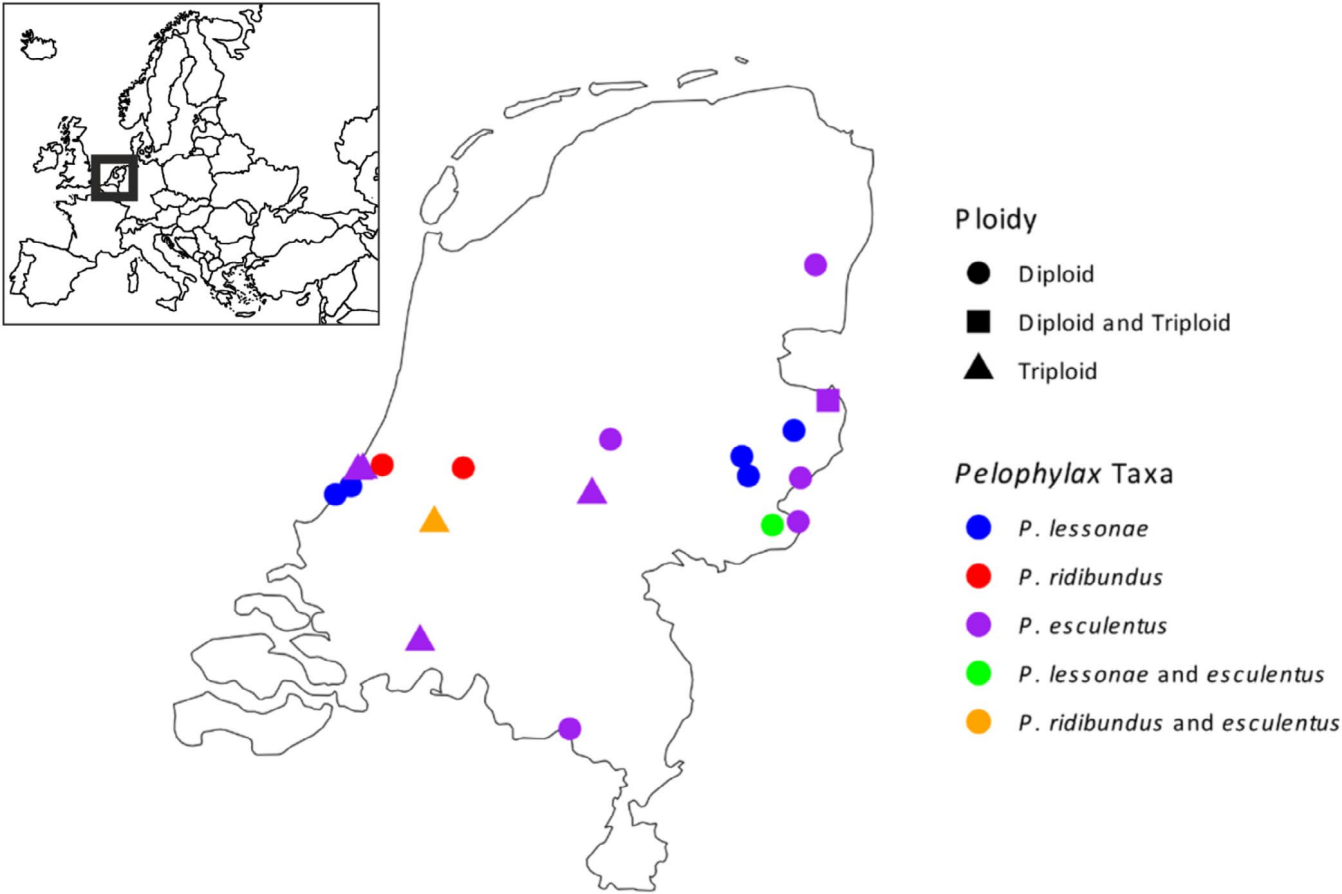
Map of waterfrog (*Pelophylax*) localities sampled in the Netherlands. *P. esculentus* occurs throughout the country, *P. ridibundus* is found in its natural range in the west. Two localities in the coastal dune areas are populated with *P. lessonae* , outside of its typical distribution in the east. All *P. esculentus* found in western areas are triploid with two copies of the *P. lessonae* genome. The sole triploid from the east instead has two copies of the *P. ridibundus* genome.

We find *P. ridibundus* samples only within the western part of the Netherlands (Figure 5), consistent with their described natural range. The majority of *P. lessonae* samples originate from the eastern Netherlands however we find that two localities within the southern part of the Dutch coastal dune areas are also populated by *P. lessonae* . We observe the presence of *P. esculentus* throughout the Netherlands, however all samples in the west are triploids with two copies of the *P. lessonae* genome, in the east all samples are diploid apart from the single triploid with two copies of the *P. ridibundus* genome.

## 4 Discussion

In this study we examine whether the target capture bait set FrogCap could be effectively used to study the *Pelophylax esculentus* hybridogenetic system, to tackle questions regarding species identification, ancestry, introgression patterns and ploidy.

The bait set used within this study (FrogCap Ranoidea v2) (Hutter et al. 2022) targets 12,909 markers, which are highly heterogeneous in terms of length (from 100 to 12,000 bp) and include exons, introns and various non‐coding ultra conserved elements. While based on transcripts from species across the Ranoidea superfamily, no sequences from *Pelophylax* were used in the bait design. Therefore, it is unsurprising that 34% of markers perform poorly in *Pelophylax*. Nevertheless, in our dataset, over 8500 marker sequences have a region of at least 100 bp with at least 10× coverage in at least 50% of individuals, sufficient for reliable phylogenetic and phylogeographic analyses.

We also note that the authors of Frogcap designed an additional bait set, Reduced‐Ranoidea (Hutter et al. 2022), that targets 3247 markers, which are a subset of the full Ranoidea set. Given that a smaller bait set is less costly and is likely to result in higher target coverage per Gbp of sequence data produced (as approximately the same number of captured reads will be spread across fewer target bases), the Reduced‐Ranoidea bait set may also be useful for investigations into *Pelophylax*.

We show that target capture data allows for clear differentiation of the taxa involved in the *P. esculentus* hybridogenetic complex. This is useful in its own right, as identification through morphology or vocalization is often difficult, especially in larvae and juveniles (Arntzen 1981; Meilink et al. 2024). Additionally, previous molecular species identification methods have proven unreliable (Dufresnes, Dubey, et al. 2024). While target capture sequencing is more resource intensive than might be desired for simple species identification, the resulting data allows for identification of a large number of species‐specific polymorphisms, which can then be used to design PCR‐based protocols, such as KASP genotyping (Semagn et al. 2014), that may prove more reliable than those currently available (Dufresnes, Dubey, et al. 2024).

We observe that polyploidy is easily recognizable using the FrogCap marker set. Compared to cytogenetic techniques or flow cytometry, the technique employed in the present study provides a simple method to determine the ploidy of a large number of samples. Furthermore, we show that it can be established which of the parental species is present in two copies. The identification of reliable species‐specific SNPs also creates the possibility of using techniques such as droplet digital PCR (Hindson et al. 2011) to identify triploids and the genome composition on a large scale. However, we note that ploidy determination in our samples was based only on allele depth ratios. While the analysis appears robust in this exploratory study, it may still be useful to validate our methodology by analyzing individuals of known ploidy through other methods.

Our data grant insight into the distribution of *Pelophylax* taxa within the Netherlands. Overall species identification matches the distribution described in the literature (Creemers and van Delft 2009). All *P. ridibundus* samples originated from localities within the west of the Netherlands, within their natural range. While *P. lessonae* is considered native to the east of the Netherlands, we confirm previous observations of populations in the coastal dune area, approximately 50 km west of their native range, and 70 km south of a previously recognized introduction in the province North Holland (NDFF 2015). Several non‐native species of amphibians are known to have been introduced to the dune area (Koster et al. 2022; Kuijt et al. 2022; Robbemont et al. 2023; Vliegenthart et al. 2022), and it is therefore likely that *P. lessonae* represents a further example.

In agreement with previous studies employing cytogenetic data (Blommers‐Schlösser 1990), we observe a marked pattern in the ploidy of *P. esculentus* samples, in the west, co‐occurring with *P. ridibundus*, all samples are triploids, with two copies of the *P. lessonae* genome. In the east, inside the natural range of *P. lessonae*, most *P. esculentus* are diploid, with only a single triploid observed (and this with the opposite configuration—two copies of the ridibundus genome). It is worth noting that our study does not include samples from the northern and central part of the range of *P. ridibundus* within the Netherlands, which would be useful to include in any future research into the population genetics of *Pelophylax*.

In a previous study (Theodoropoulos et al. 2025) we observed that *P. lessonae* mtDNA was extremely widespread throughout *Pelophylax* samples throughout the Netherlands, regardless of which *Pelophylax* taxon the sample was assigned to. Our results in this study suggest that this is due to mtDNA introgression of *P. lessonae* mtDNA into *P. ridibundus* via *P. esculentus* . Except for a single *P. esculentus* , all individuals, including all *P. ridibundus* , possess *P. lessonae* mtDNA. Unidirectional introgression of mtDNA is the expected result of the *lessonae*‐eliminating system present in the Netherlands. As *P. esculentus* typically mate with *P. lessonae* generation after generation, it is highly likely that they will eventually inherit *P. lessonae* mtDNA. When a *P. esculentus* female carrying *P. lessonae* mtDNA mates with *P. ridibundus* , the result will be an individual with purely *P. ridibundus* nuclear DNA but *P. lessonae* mtDNA (Plötner et al. 2008).

We also found that several *P. esculentus* samples appear to have slightly greater than 50% *P. lessonae* ancestry. This could potentially be seen as evidence of nuclear gene flow (Mezhzherin et al. 2024; Mikulíček et al. 2014; Uzzell et al. 1976), especially as the mechanics of the hybridogenetic system mean that any introgression of nuclear DNA is likely to follow the same pattern as mtDNA, against the direction of genome elimination. However, we propose this is more likely to be an artifact of the unequal allele ratios present in triploids, resulting in heterozygous haplotypes occasionally being miscalled as homozygous for the parental species contributing twice the copy number (a known complication in polyploids; e.g., Wang et al. 2024). The affected samples, deriving from multiple localities, are all triploids with two copies of the *P. lessonae* genome. Furthermore, the two samples with the largest excess *P. lessonae* contribution are the two triploids with the lowest overall coverage, and therefore the individuals most likely to be affected by miscalled haplotypes where the *P. ridibundus* allele is not recorded.

While *P. ridibundus* and *P. lessonae* are the only ancestral lineages natively present within the Netherlands (and hence examined in this study), the genus *Pelophylax* includes other taxa capable of forming hybridogenetic complexes, for example the Iberian frog *P. grafi*, a hybridogenetic hybrid between *P. perezi* and *P. ridibundus* (Graf et al. 1977). The interactions between these taxa are less well studied, especially when their native ranges do not border each other. However, due to the large number of anthropogenic *Pelophylax* introductions in recent times (Denoël and Dufresnes 2025; Doniol‐Valcroze et al. 2021; Dufresnes and Dubey 2020; Papežík et al. 2024), these exotic systems may become important. The ability to exclude a competing genome from gametogenesis could allow invasive lineages to spread through, and displace, native populations extremely rapidly. Consequently, *Pelophylax* has been identified as one of the taxa most vulnerable to disruption of native species by introduced congeners, creating a critical need for monitoring (Dufresnes and Mazepa 2020).

Unfortunately, it is not always easy to determine whether an introduction has taken place, given the variable morphology of *Pelophylax*, and limitations of current molecular approaches. The target capture approach we test in this study offers a powerful tool for examining the ancestral contribution of many lineages to any given population. A possible example of interest is the sample NL_1673, which appears highly divergent from other *P. lessonae* samples in the PCA. As this divergence was orthogonal to the otherwise dominant *P. lessonae* — *P. ridibundus* variation, it cannot easily be explained by interaction between the native taxa. However, this divergence may represent a non‐native *P. lessonae* individual. As exemplified by the non‐native coastal dune population (see above), as well as the presence of non‐native mtDNA elsewhere in the Netherlands (Theodoropoulos et al. 2025), *P. lessonae* has been moved around by humans. While the sample NL_1673 carries an mtDNA haplotype that is naturally found in the Netherlands, the distribution range of this haplotype covers a considerably larger geographical area, from Norway to Russia to the Ukraine to Italy (Theodoropoulos et al. 2025). The hypothesis that sample NL_1673 concerns a *P. lessonae* individual of foreign origin could be tested by applying the target capture methodology to a wider selection of *Pelophylax* samples.

We conclude that target capture sequencing employing the FrogCap marker set (Hutter et al. 2022) is an effective method of gathering large‐scale genetic information from samples within the genus *Pelophylax*. We recommend its use for future studies investigating the hybridogenetic complexes and other evolutionary and ecological questions in the genus.

## Author Contributions

**Lisanne van Veldhuijzen:** formal analysis (lead), investigation (lead), writing – original draft (equal), writing – review and editing (equal). **Maciej Pabijan:** resources (equal), writing – review and editing (equal). **Tariq Stark:** resources (equal), writing – review and editing (equal). **Richard P. J. H. Struijk:** resources (equal), writing – review and editing (equal). **Ben Wielstra:** conceptualization (equal), resources (equal), supervision (supporting), writing – review and editing (equal). **James France:** conceptualization (equal), formal analysis (supporting), supervision (lead), writing – original draft (equal), writing – review and editing (equal).

## Conflicts of Interest

The authors declare no conflicts of interest.

## Supporting information


**Figure S1:** Ploidy of *Pelophylax ridibundus* samples. In each subfigure, the most likely model of ploidy and the *R* ^2^ value for the best ploidy model are noted. The x‐axis displays the allele ratio of each SNP. The y‐axis shows number of SNPs with a given allele ratio. All *P. ridibundus* samples were found to be diploid.
**Figure S2:** Ploidy of *Pelophylax lessonae* samples. In each subfigure, the most likely model of ploidy and the *R* ^2^ value for the best ploidy model are noted. The x‐axis displays the allele ratio of each SNP. The y‐axis shows number of SNPs with a given allele ratio. All *P. lessonae* samples were found to be diploid.
**Figure S3:** Ploidy of *Pelophylax esculentus* samples. In each subfigure, the most likely model of ploidy and the *R* ^2^ value for the best ploidy model are noted. The x‐axis displays the allele ratio of each SNP. The y‐axis shows number of SNPs with a given allele ratio. 9 *P. esculentus* samples were found to be triploid and the rest diploid.
**Table S1:** Details of samples used in this study.

## Data Availability

All raw reads can be found as a part of the NCBI accession associated with Bioproject: PRJNA1330369. All scripts used for the processing of data in this study are available at the project's GitHub repository: https://github.com/Wielstra‐Lab/Pelophylax_target_capture—a permanent code is available via Zenodo: https://doi.org/10.5281/zenodo.19693367.
