# Supplementary material for: Target Capture Sequencing Provides Insights Into Hybridogenetic Water Frogs
Source: Ecol Evol. 2026 Apr 29;16(5):e73570. doi: 10.1002/ece3.73570 (PMC13125954; doi:10.1002/ece3.73570)
Supplement: Supplementary file 1 — Figure S1: Ploidy of Pelophylax ridibundus samples. In each subfigure, the most likely model of ploidy and the R 2 value for the best ploidy model are noted. The x‐axis displays the allele ratio of each SNP. The y‐axis shows number of SNPs with a given allele ratio. All P. ridibundus samples were found to be diploid. Figure S2: Ploidy of Pelophylax lessonae samples. In each subfigure, the most likely model of ploidy and the R 2 value for the best ploidy model are noted. The x‐axis displays the allele ratio of each SNP. The y‐axis shows number of SNPs with a given allele ratio. All P. lessonae samples were found to be diploid. Figure S3: Ploidy of Pelophylax esculentus samples. In each subfigure, the most likely model of ploidy and the R 2 value for the best ploidy model are noted. The x‐axis displays the allele ratio of each SNP. The y‐axis shows number of SNPs with a given allele ratio. 9 P. esculentus samples were found to be triploid and the rest diploid. Table S1: Details of samples used in this study. [file ECE3-16-e73570-s001.docx]

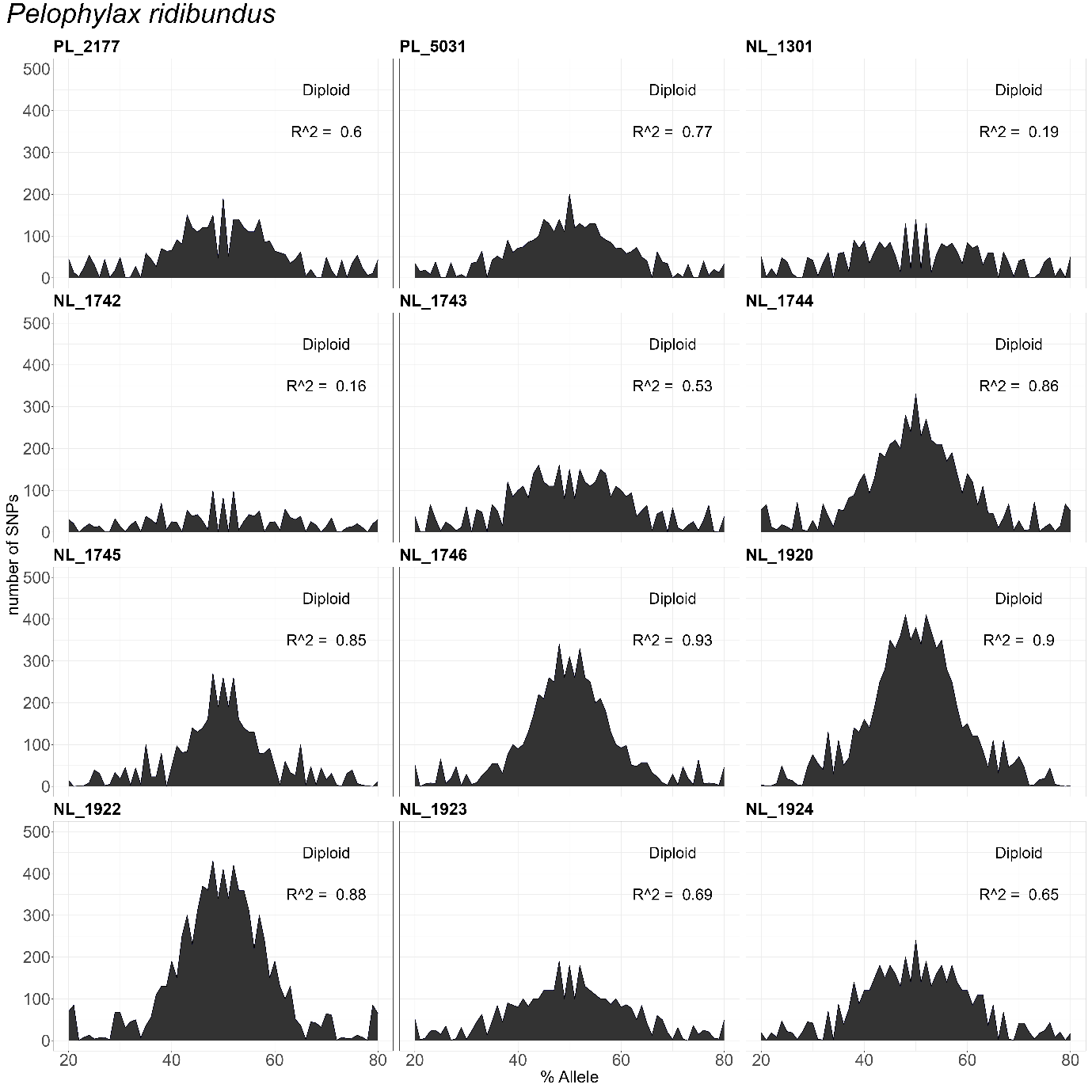


**Fig S1.** **Ploidy of *Pelophylax ridibundus* samples**. In each subfigure, the most likely model of ploidy and the R^2^ value for the best ploidy model are noted. The x-axis displays the allele ratio of each SNP. The y-axis shows number of SNPs with a given allele ratio. All *P. ridibundus* samples were found to be diploid.


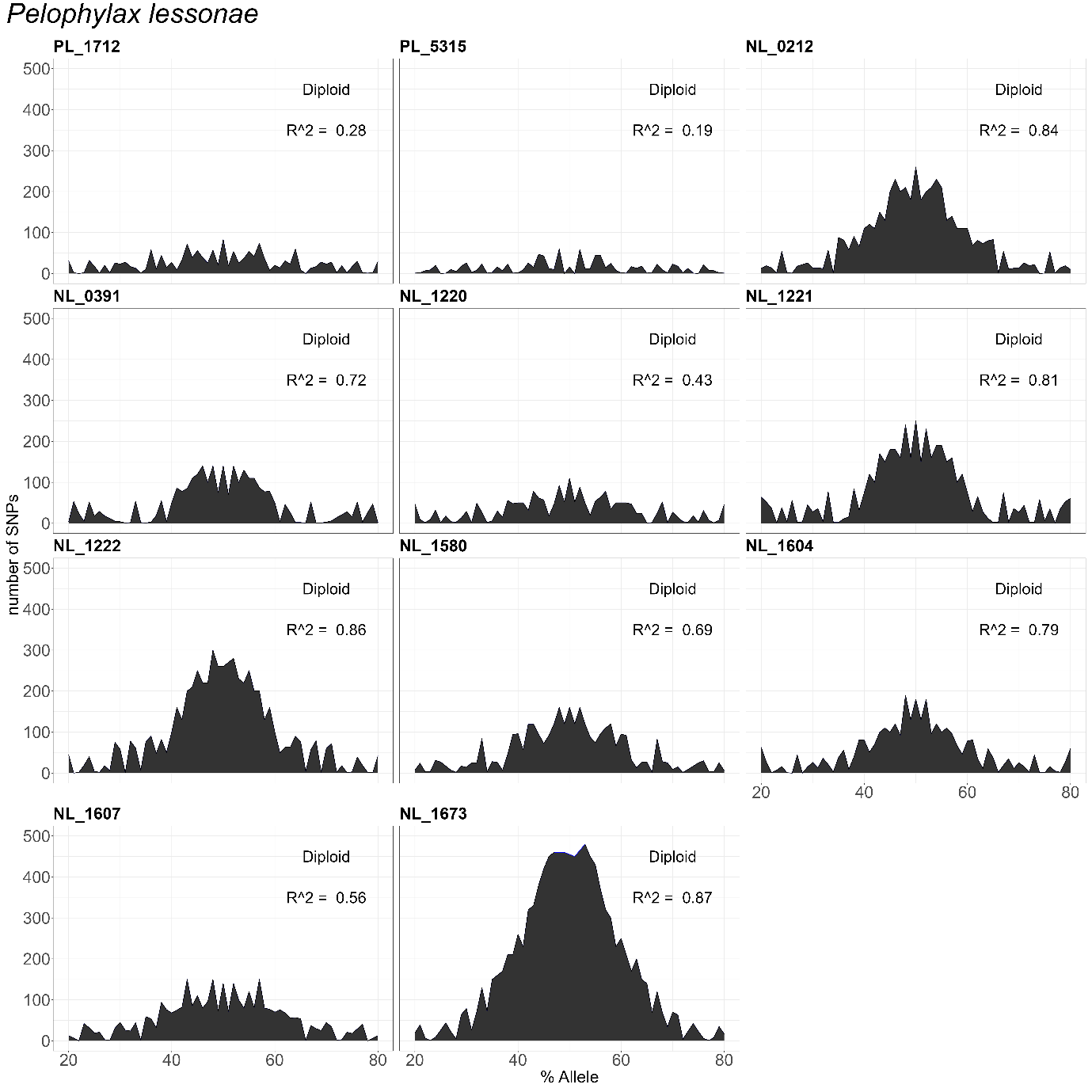


**Fig S2.** **Ploidy of *Pelophylax lessonae* samples**. In each subfigure, the most likely model of ploidy and the R^2^ value for the best ploidy model are noted. The x-axis displays the allele ratio of each SNP. The y-axis shows number of SNPs with a given allele ratio. All *P. lessonae* samples were found to be diploid.


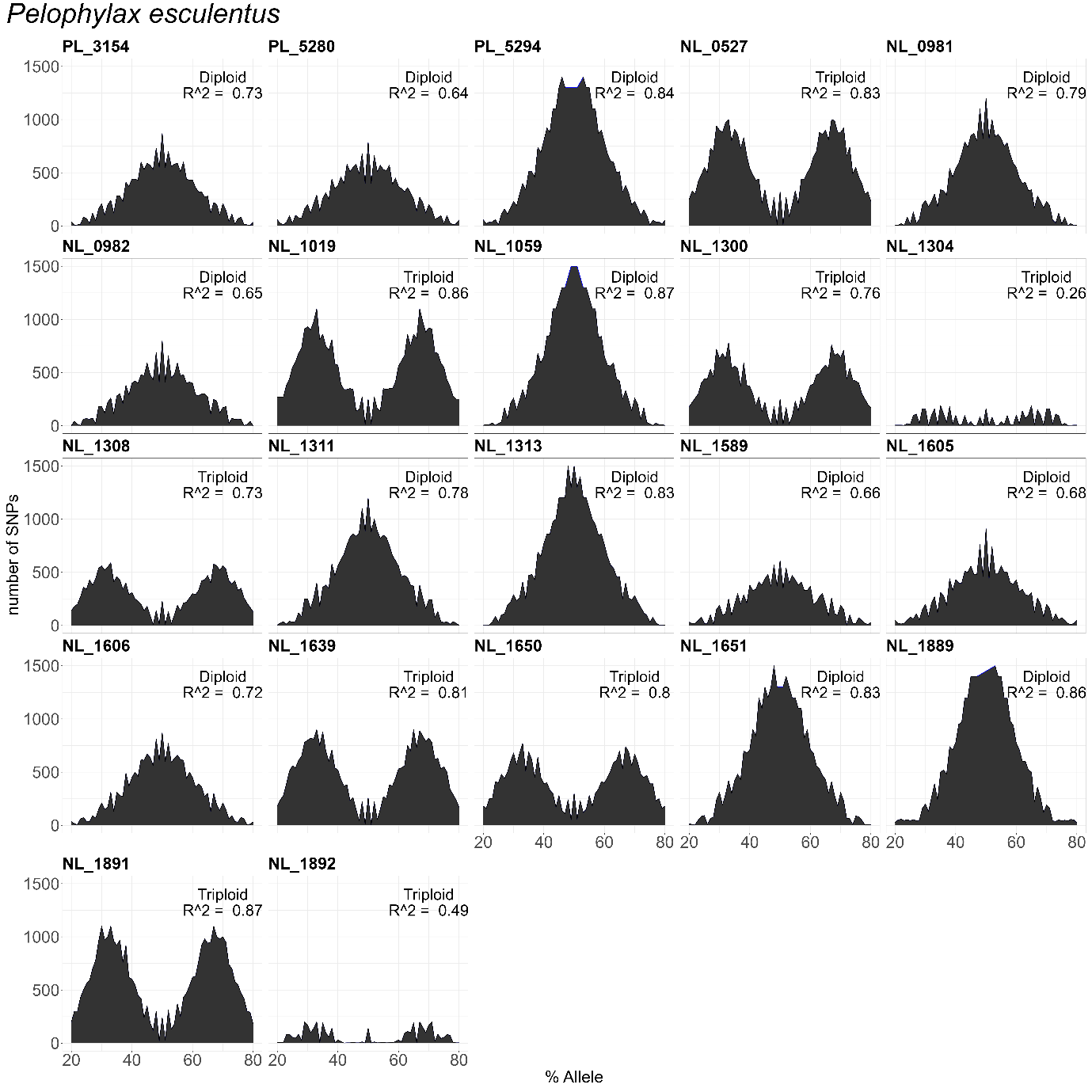


**Fig S3.** **Ploidy of *Pelophylax esculentus* samples**. In each subfigure, the most likely model of ploidy and the R^2^ value for the best ploidy model are noted. The x-axis displays the allele ratio of each SNP. The y-axis shows number of SNPs with a given allele ratio. 9 *P. esculentus* samples were found to be triploid and the rest diploid.

**Table S1.** **Details of samples used in this study**.

| **Sample ID** | **Alternative ID** | **Sample type** | **Country** | **Locality** | **Latitude** | **Longitude** | **mtDNA** | **Taxon (PCA)** | **Ploidy** | **Copy number** | | **SRA accession number** |
| --- | --- | --- | --- | --- | --- | --- | --- | --- | --- | --- | --- | --- |
|  |  |  |  |  |  |  |  |  |  | ***P. lessonae*** | ***P. ridibundus*** |  |
| NL_0212 | swab_0212 | Buccal swab | Netherlands | Meijendel TNO | 52.1114 | 4.3242 | *P. lessonae* | *P. lessonae* | Diploid | 2 | 0 | SRR35489742 |
| NL_0391 | swab_0391 | Skin swab | Netherlands | Den Haag, Westduinpark | 52.0833 | 4.2393 | *P. lessonae* | *P. lessonae* | Diploid | 2 | 0 | SRR35489760 |
| NL_0527 | swab_0527 | Buccal swab | Netherlands | Liesbos | 51.5866 | 4.7047 | *P. ridibundus* | *P. esculentus* | Triploid | 2 | 1 | SRR35489747 |
| NL_0981 | swab_0981 | Buccal swab | Netherlands | Valthe | 52.8571 | 6.8809 | *P. lessonae* | *P. esculentus* | Diploid | 1 | 1 | SRR35489729 |
| NL_0982 | swab_0982 | Buccal swab | Netherlands | Valthe | 52.8503 | 6.8827 | *P. lessonae* | *P. esculentus* | Diploid | 1 | 1 | SRR35489727 |
| NL_1019 | swab_1019 | Buccal swab | Netherlands | Duinoord | 52.1630 | 4.3637 | *P. lessonae* | *P. esculentus* | Triploid | 2 | 1 | SRR35489756 |
| NL_1059 | swab_1059 | Tissue | Netherlands | Haaksbergerveen | 52.1387 | 6.7984 | *P. lessonae* | *P. esculentus* | Diploid | 1 | 1 | SRR35489753 |
| NL_1220 | swab_1220 | Skin swab | Netherlands | Den Haag, Westduinpark | 52.0833 | 4.2393 | *P. lessonae* | *P. lessonae* | Diploid | 2 | 0 | SRR35489759 |
| NL_1221 | swab_1221 | Skin swab | Netherlands | Den Haag, Westduinpark | 52.0833 | 4.2393 | *P. lessonae* | *P. lessonae* | Diploid | 2 | 0 | SRR35489758 |
| NL_1222 | swab_1222 | Skin swab | Netherlands | Den Haag, Westduinpark | 52.0833 | 4.2393 | *P. lessonae* | *P. lessonae* | Diploid | 2 | 0 | SRR35489757 |
| NL_1300 | swab_1300 | Buccal swab | Netherlands | Bilwijk Krimpenerwaard | 51.9871 | 4.7836 | *P. lessonae* | *P. esculentus* | Triploid | 2 | 1 | SRR35489720 |
| NL_1301 | swab_1301 | Buccal swab | Netherlands | Bilwijk Krimpenerwaard | 51.9871 | 4.7836 | *P. lessonae* | *P. ridibundus* | Diploid | 0 | 2 | SRR35489719 |
| NL_1304 | swab_1304 | Buccal swab | Netherlands | Lentevreugd | 52.1658 | 4.3900 | *P. lessonae* | *P. esculentus* | Triploid | 2 | 1 | SRR35489749 |
| NL_1308 | swab_1308 | Buccal swab | Netherlands | Lentevreugd | 52.1658 | 4.3900 | *P. lessonae* | *P. esculentus* | Triploid | 2 | 1 | SRR35489748 |
| NL_1311 | swab_1311 | Buccal swab | Netherlands | Gastelsche Heide | 51.2930 | 5.5289 | *P. lessonae* | *P. esculentus* | Diploid | 1 | 1 | SRR35489755 |
| NL_1313 | swab_1313 | Buccal swab | Netherlands | Gastelsche Heide | 51.2930 | 5.5289 | *P. lessonae* | *P. esculentus* | Diploid | 1 | 1 | SRR35489754 |
| NL_1580 | swab_1580 | Tissue | Netherlands | Wanninkhof | 52.1456 | 6.5114 | *P. lessonae* | *P. lessonae* | Diploid | 2 | 0 | SRR35489723 |
| NL_1589 | swab_1589 | Skin swab | Netherlands | Masterveld | 51.9918 | 6.7858 | *P. lessonae* | *P. esculentus* | Diploid | 1 | 1 | SRR35489743 |
| NL_1604 | swab_1604 | Tissue | Netherlands | Visschersdijk | 52.2115 | 6.4758 | *-* | *P. lessonae* | Diploid | 2 | 0 | SRR35489726 |
| NL_1605 | swab_1605 | Tissue | Netherlands | Haaksbergerveen | 52.1196 | 6.7837 | *P. lessonae* | *P. esculentus* | Diploid | 1 | 1 | SRR35489752 |
| NL_1606 | swab_1606 | Tissue | Netherlands | Vragenderveen | 51.9798 | 6.6441 | *-* | *P. esculentus* | Diploid | 1 | 1 | SRR35489725 |
| NL_1607 | swab_1607 | Tissue | Netherlands | Vragenderveen | 51.9798 | 6.6441 | *P. lessonae* | *P. lessonae* | Diploid | 2 | 0 | SRR35489724 |
| NL_1639 | swab_1639 | Buccal swab | Netherlands | Lunterse Buurtbos | 52.0832 | 5.6513 | *P. lessonae* | *P. esculentus* | Triploid | 2 | 1 | SRR35489746 |
| NL_1650 | swab_1650 | Skin swab | Netherlands | Plooi Tilligte | 52.4000 | 6.9500 | *P. lessonae* | *P. esculentus* | Triploid | 1 | 2 | SRR35489741 |
| NL_1651 | swab_1651 | Skin swab | Netherlands | Plooi Tilligte | 52.4000 | 6.9500 | *P. lessonae* | *P. esculentus* | Diploid | 1 | 1 | SRR35489740 |
| NL_1673 | swab_1673 | Skin swab | Netherlands | Borne | 52.2986 | 6.7625 | *P. lessonae* | *P. lessonae* | Diploid | 2 | 0 | SRR35489718 |
| NL_1742 | swab_1742 | Skin swab | Netherlands | Kockengen | 52.1728 | 4.9425 | *P. lessonae* | *P. ridibundus* | Diploid | 0 | 2 | SRR35489734 |
| NL_1743 | swab_1743 | Skin swab | Netherlands | Kockengen | 52.1728 | 4.9425 | *P. lessonae* | *P. ridibundus* | Diploid | 0 | 2 | SRR35489733 |
| NL_1744 | swab_1744 | Skin swab | Netherlands | Kockengen | 52.1728 | 4.9425 | *P. lessonae* | *P. ridibundus* | Diploid | 0 | 2 | SRR35489732 |
| NL_1745 | swab_1745 | Skin swab | Netherlands | Kockengen | 52.1728 | 4.9425 | *P. lessonae* | *P. ridibundus* | Diploid | 0 | 2 | SRR35489731 |
| NL_1746 | swab_1746 | Skin swab | Netherlands | Kockengen | 52.1728 | 4.9425 | *P. lessonae* | *P. ridibundus* | Diploid | 0 | 2 | SRR35489730 |
| NL_1889 | swab_1889 | Buccal swab | Netherlands | Landgoed Staverden | 52.2688 | 5.7526 | *P. lessonae* | *P. esculentus* | Diploid | 1 | 1 | SRR35489751 |
| NL_1891 | swab_1891 | Buccal swab | Netherlands | Lunterse Buurtbos | 52.0867 | 5.6513 | *P. lessonae* | *P. esculentus* | Triploid | 2 | 1 | SRR35489745 |
| NL_1892 | swab_1892 | Buccal swab | Netherlands | Lunterse Buurtbos | 52.0867 | 5.6513 | *P. lessonae* | *P. esculentus* | Triploid | 2 | 1 | SRR35489744 |
| NL_1920 | swab_1920 | Buccal swab | Netherlands | Poelgeest | 52.1832 | 4.4972 | *P. lessonae* | *P. ridibundus* | Diploid | 0 | 2 | SRR35489738 |
| NL_1922 | swab_1922 | Buccal swab | Netherlands | Poelgeest | 52.1832 | 4.4972 | *P. lessonae* | *P. ridibundus* | Diploid | 0 | 2 | SRR35489737 |
| NL_1923 | swab_1923 | Tissue | Netherlands | Poelgeest | 52.1832 | 4.4972 | *P. lessonae* | *P. ridibundus* | Diploid | 0 | 2 | SRR35489736 |
| NL_1924 | swab_1924 | Buccal swab | Netherlands | Poelgeest | 52.1832 | 4.4972 | *P. lessonae* | *P. ridibundus* | Diploid | 0 | 2 | SRR35489735 |
| PL_1712 | MPFC1712 | Tissue | Poland | Klewiny | 54.2834 | 22.0877 | *P. lessonae* | *P. lessonae* | Diploid | 2 | 0 | SRR35489762 |
| PL_2177 | MPFC2177 | Tissue | Poland | Stawy Bugaj | 49.9813 | 19.4250 | *P. lessonae* | *P. ridibundus* | Diploid | 0 | 2 | SRR35489739 |
| PL_3154 | MPFC3154 | Tissue | Poland | Przeręb | 50.0145 | 19.4056 | *P. lessonae* | *P. esculentus* | Diploid | 1 | 1 | SRR35489728 |
| PL_5031 | MPFC5031 | Tissue | Poland | Mydlniki | 50.0843 | 19.8403 | *P. ridibundus* | *P. ridibundus* | Diploid | 0 | 2 | SRR35489750 |
| PL_5280 | MPFC5280 | Tissue | Poland | Przeręb | 50.0145 | 19.4056 | *P. lessonae* | *P. esculentus* | Diploid | 1 | 1 | SRR35489722 |
| PL_5294 | MPFC5294 | Tissue | Poland | Ispina | 50.1132 | 20.3581 | *P. lessonae* | *P. esculentus* | Diploid | 1 | 1 | SRR35489721 |
| PL_5315 | MPFC5315 | Tissue | Poland | Straż | 53.3395 | 23.3711 | *P. lessonae* | *P. lessonae* | Diploid | 2 | 0 | SRR35489761 |
